# Analgesic Efficacy of Bilateral Superficial Cervical Plexus Block Using Levobupivacaine vs Ropivacaine for Thyroid Surgery Under General Anesthesia

**DOI:** 10.7759/cureus.112386

**Published:** 2026-07-10

**Authors:** Velmurugananth Velayutham, Ramanikanth Subramaniam, Rathnakumari Udayakumar, Arunagiri Gunasekar, Manoranjini Venkatesan

**Affiliations:** 1 Department of Anesthesiology, Government Kilpauk Medical College, Chennai, IND; 2 Department of Anesthesiology, Government Medical College, Vellore, IND; 3 Department of Physiology, Government Kilpauk Medical College, Chennai, IND; 4 Department of Anesthesiology, Government Medical College, Thiruvallur, IND

**Keywords:** analgesic efficacy, general anesthesia, superficial cervical plexus block, thyroidectomy, thyroid surgery

## Abstract

Background

Thyroidectomy is commonly associated with moderate postoperative pain, which may delay recovery and discharge. Bilateral superficial cervical plexus block (BSCPB) is an established component of multimodal analgesia for thyroid surgery. Levobupivacaine and ropivacaine are long-acting aminoamide local anesthetics with improved safety over bupivacaine, but comparative data on their efficacy for BSCPB in thyroidectomy remain limited.

Aim

This study aimed to compare, in a real-world government teaching hospital cohort, the postoperative analgesic efficacy and duration of analgesia of 0.25% levobupivacaine vs 0.25% ropivacaine when used for landmark-guided BSCPB in patients undergoing thyroid surgery under general anesthesia, an institution-specific comparison for which local, technique-specific comparative data were previously unavailable.

Materials and methods

This prospective, observational, comparative study was conducted in the Department of Anesthesiology, Government Medical College and Hospital, Chengalpattu. Sixty American Society of Anesthesiologists I-II patients aged 20-55 years scheduled for elective thyroidectomy under general anesthesia were enrolled into two groups (n = 30 each): Group L received a total of 12 mL of 0.25% levobupivacaine (6 mL per side), and Group R received a total of 12 mL of 0.25% ropivacaine (6 mL per side) for landmark-guided BSCPB after induction. The primary outcome was the duration of analgesia (time from block to first rescue analgesic at a Visual Analog Scale (VAS) ≥4). Secondary outcomes included postoperative VAS scores at one, four, eight, 12, 16, 20, and 24 hours; the number of rescue analgesic doses in 24 hours; hemodynamic profile; and adverse events.

Results

Demographic and baseline variables were comparable between the groups. The duration of analgesia was significantly longer in Group L than in Group R (549.3 ± 36.3 vs 370.0 ± 59.8 minutes; p < 0.001). Postoperative VAS scores at 4, 8, 12, 16, 20, and 24 hours were significantly lower in Group L (p ≤ 0.007). A smaller proportion of patients in Group L required rescue analgesia compared with Group R (40% vs 80%; p = 0.002), and total 24-hour paracetamol consumption was significantly lower in Group L. Both groups remained hemodynamically stable, and no serious block-related complications (defined as hematoma requiring intervention, phrenic nerve palsy with respiratory compromise, or features of local anesthetic systemic toxicity) were observed. Non-serious, self-limiting events (postoperative nausea and vomiting and mild sore throat) occurred at low and comparable rates in both groups.

Conclusions

In this comparative observational study, BSCPB with 0.25% levobupivacaine provided a significantly longer duration of analgesia and lower postoperative VAS pain scores than 0.25% ropivacaine in thyroid surgery, indicating superior analgesic efficacy as measured by these validated, prespecified outcome measures, with reduced rescue analgesic requirements and a similar safety profile. Levobupivacaine is preferable to ropivacaine for BSCPB in thyroidectomy under general anesthesia.

## Introduction

Thyroidectomy is a frequently performed head and neck procedure, and multiple contemporary series report same-day or short-stay (23-hour) discharge in 30-70% of eligible patients undergoing uncomplicated thyroidectomy [1,2], making optimized postoperative analgesia essential for early recovery and discharge. Postoperative pain after thyroid surgery is typically of mild to moderate intensity but can significantly impair swallowing, neck movement, and early mobilization if not adequately treated [1,3]. Inadequate pain control is one of several recognized contributors to postoperative nausea and vomiting (PONV), alongside opioid use, anesthetic technique, and patient-related risk factors, and is also associated with delayed discharge and reduced patient satisfaction [3,4]. This study was not designed to isolate the independent contribution of pain control to PONV. Systemic analgesics such as opioids and nonsteroidal anti-inflammatory drugs remain the mainstay of postoperative pain management, but their usefulness is limited by dose-dependent adverse effects, including respiratory depression, sedation, PONV, pruritus, ileus, bleeding, and renal dysfunction [4-6]. These limitations have driven interest in opioid-sparing multimodal analgesia and regional anesthetic techniques to improve pain control while minimizing systemic side effects [5,6].

Bilateral superficial cervical plexus block (BSCPB) is a simple regional technique that anesthetizes the superficial branches of the cervical plexus supplying the anterolateral neck and thyroidectomy incision area [7]. When combined with general anesthesia, BSCPB has been shown in randomized trials and meta-analyses to reduce postoperative pain scores, opioid requirements, and early rescue analgesic use after thyroidectomy [1,2,8,9]. These properties make BSCPB an attractive component of multimodal and enhanced recovery pathways for thyroid surgery [8,9].

Levobupivacaine and ropivacaine are long-acting aminoamide local anesthetics developed as less cardiotoxic and less neurotoxic alternatives to racemic bupivacaine [10,11]. Levobupivacaine, the S(-)-enantiomer of bupivacaine, is more lipophilic and highly protein-bound than ropivacaine, features that are associated with prolonged sensory block, whereas ropivacaine is slightly less lipophilic than levobupivacaine and exhibits more favorable motor-sensory differentiation [11,12]. Meta-analytic and clinical data on peripheral nerve blocks suggest that levobupivacaine may confer a modestly longer duration of sensory block and reduce the need for rescue analgesia compared with ropivacaine, while both agents maintain comparable safety profiles [12,13]. Therefore, this prospective, observational, comparative study was conducted to compare the analgesic efficacy, duration of analgesia, and safety of 0.25% levobupivacaine vs 0.25% ropivacaine for BSCPB in patients undergoing elective thyroid surgery under general anesthesia.

## Materials and methods

Study design and setting

This prospective, observational, comparative study was conducted in the Department of Anesthesiology, Government Chengalpattu Medical College and Hospital, Tamil Nadu, India, from January 2021 to December 2021, after obtaining approval from the Institutional Ethics Committee (approval no. ECR/774/INST/TN/2015) and written informed consent from all participants. The study conformed to the principles of the Declaration of Helsinki for research involving human subjects.

Study population

For planning purposes, the sample size for this study was estimated a priori using published pilot data on the primary outcome of postoperative Visual Analog Scale (VAS) pain score at four hours [14-17], recognizing that this is an approximation rather than a confirmed effect size for the present landmark-guided technique. A previous prospective, randomized, double-blind study by Rajeswari et al. comparing ultrasound-guided superficial cervical plexus block with 0.5% bupivacaine and 0.5% ropivacaine in patients undergoing thyroid surgery reported mean VAS pain scores at the four-hour postoperative interval of 2.5 ± 0.9 and 2.4 ± 0.8, respectively [17]. Using these data, the pooled SD was calculated as σ = 0.85. As the reference study used an ultrasound-guided technique and higher local anesthetic concentrations (0.5%) than the present landmark-guided study (0.25%), the derived variance estimate was considered an approximation for a priori feasibility planning rather than a precise estimate of the expected effect size. Assuming a clinically meaningful difference (Δ) of 0.65 VAS units between the 0.25% levobupivacaine and 0.25% ropivacaine groups, a two-sided significance level (α) of 0.05 (Zα/2 = 1.96), and a statistical power of 80% (β = 0.20; Zβ = 0.84), the estimated sample size per group was calculated using the independent-samples t-test formula [17]:

\(n = \frac{2\left(Z_{\alpha/2}+Z_{\beta}\right)^2\sigma^2}{\Delta^2} = \frac{2(1.96+0.84)^2(0.85)^2}{(0.65)^2} \approx 26 \text{ patients per group}\)

To account for potential dropouts and maintain feasibility within the institutional setting, 30 patients were enrolled in each group (60 total). This sample size provides 80% statistical power to detect a minimum clinically meaningful difference of 0.61 VAS units (Cohen’s d = 0.59) in postoperative pain scores between the two local anesthetic groups.

Patient enrollment and group assignment

Patients were enrolled sequentially based on availability and surgical scheduling. The first 30 consecutive patients presenting to the department were allocated to Group L (levobupivacaine), and the subsequent 30 consecutive patients were allocated to Group R (ropivacaine). This was a sequential, nonrandomized allocation based strictly on the order of presentation, and no randomization procedure (e.g., a computer-generated sequence or random number table) was used to form the two study groups. Patients were informed of the local anesthetic being used, but postoperative pain assessments were performed by trained ward nursing staff, and statistical analyses were performed by data analysts, both of whom were blinded to group assignment.

Anesthetic technique

All patients were admitted as inpatients the evening before surgery, fasted for a minimum of eight hours for solids and two hours for clear fluids in accordance with the American Society of Anesthesiologists (ASA) preoperative fasting guidelines [18], and received oral ranitidine 150 mg and alprazolam 0.25 mg the night before surgery. In the operating room, baseline heart rate (HR), noninvasive blood pressure (NIBP), and peripheral oxygen saturation (SpO₂) were recorded [18]. After establishing IV access, patients were premedicated with IV midazolam 0.02 mg/kg and fentanyl 2 μg/kg. Standard monitoring (ECG, NIBP, SpO₂, and end-tidal CO₂) was applied. General anesthesia was induced with propofol 2 mg/kg and rocuronium 0.6 mg/kg to facilitate tracheal intubation. Anesthesia was maintained with a mixture of oxygen and air and sevoflurane, titrated to a target end-tidal concentration corresponding to 1.0-1.5 age-adjusted minimum alveolar concentration equivalents for each patient, together with intermittent doses or an infusion of rocuronium as required. End-tidal sevoflurane concentration (vol%) was continuously displayed and adjusted individually and was not fixed at a single absolute dose across all patients. Ventilation was controlled to maintain normocapnia.

BSCPB technique

After induction and securing the airway, with the patient in the supine position and the head slightly turned to the opposite side, the landmark-guided BSCPB was performed by an experienced anesthesiologist (>5 years of experience in regional anesthesia). The posterior border of the sternocleidomastoid (SCM) muscle at the junction of its upper two-thirds and lower one-third was identified on each side. Using a 23-G short-bevel needle, the following three-point subcutaneous injection technique was used on each side (total volume, 6 mL per side): (1) Midpoint injection (2 mL): At the midpoint of the posterior border of the SCM, 2 mL of the study drug was injected subcutaneously after negative aspiration and spread cephalad and caudad along the posterior border. (2) Medial injection (2 mL): The needle was redirected slightly medially and advanced just anterior to the posterior border of the SCM. Two milliliters of the drug were injected after careful aspiration. (3) Supraclavicular injection (2 mL): At the level just above the clavicle, 2 mL of the drug was injected subcutaneously to block the supraclavicular branches.

Thus, a total of 12 mL of the assigned local anesthetic (6 mL on each side) was administered to each patient [18-22]. Each side was injected sequentially, with continuous pulse oximetry and respiratory monitoring (SpO₂, respiratory rate, and clinical observation for dyspnea or diaphragmatic splinting) maintained between the first and second side injections to detect early signs of phrenic nerve involvement before proceeding to the contralateral side. No clinical evidence of hemidiaphragmatic paresis was observed in any patient.

Study groups

Group L (levobupivacaine group, n = 30) received 12 mL (6 mL per side) of 0.25% levobupivacaine for BSCPB, and Group R (ropivacaine group, n = 30) received 12 mL (6 mL per side) of 0.25% ropivacaine for BSCPB. The study drug was prepared by an anesthesiologist not involved in data collection and provided in identical syringes labeled with the patient’s initials and study number.

Intraoperative monitoring and management

Standard intraoperative monitoring, including ECG, NIBP, SpO₂, and end-tidal carbon dioxide, was used in accordance with the ASA Standards for Basic Intraoperative Monitoring [14]. Hemodynamic parameters, including HR, systolic blood pressure (SBP), diastolic blood pressure (DBP), and mean arterial pressure (MAP), were recorded at baseline, post-induction, post-intubation, at 15, 30, and 45 minutes, and at the end of surgery. Episodes of hypotension (SBP <90 mmHg or >20% decrease from baseline) were treated with a fluid bolus and vasopressors as needed. Bradycardia (HR <50/min) was treated with atropine 0.6 mg IV. No additional regional technique was used. Intraoperative fentanyl supplementation was administered only if HR or SBP increased by >20% from baseline in response to surgical stimulation, and total fentanyl use was recorded.

Postoperative analgesia and assessment

After extubation, patients were transferred to the postanesthesia care unit (PACU) and later to the ward. The duration of analgesia was defined operationally as the time interval, in minutes, from completion of the BSCPB to the first request for rescue analgesia (VAS ≥4), as identified by the blinded nursing assessor. No additional provocative sensory testing (e.g., pinprick or cold testing) was performed to verify block regression, and the time to first analgesic request was used as a validated surrogate for the duration of analgesia, consistent with previous BSCPB literature [1,2,8]. Pain intensity was assessed using a standard 10-cm VAS (0 = no pain, 10 = worst imaginable pain) at one, four, eight, 12, 16, 20, and 24 hours postoperatively by nursing staff blinded to group allocation. The identity of the study drug was not recorded in the intraoperative anesthesia or procedure notes accessible to the assessing nurses, and patients were instructed not to disclose the drug name to the postoperative assessment team to preserve assessor blinding. Rescue analgesia was standardized as follows: IV paracetamol 1 g was administered when VAS was ≥4 (assessed at the specified time points and on an as-needed basis whenever a patient reported breakthrough pain). If the VAS score remained >4 after 30 minutes, IV tramadol 1 mg/kg was administered [23].

Statistical analysis

Data were analyzed using IBM SPSS Statistics for Windows, version 30.0 (released 2024; IBM Corp., Armonk, NY, USA). Continuous variables were expressed as mean ± SD and compared using an independent-samples t-test. Categorical variables were expressed as numbers and percentages and compared using the chi-square test or Fisher’s exact test, as appropriate. Repeated-measures ANOVA was used to assess pain scores over time between groups. A p-value <0.05 was considered statistically significant.

## Results

Demographic and baseline characteristics

All 60 enrolled patients completed the study and were included in the analysis. There were no statistically significant differences between the groups in age, sex distribution, body weight, BMI, ASA physical status, or baseline hemodynamic parameters. Table 1 shows the demographics and baseline characteristics. 

**Table 1:** Demographic and baseline characteristics Demographic and baseline characteristics comparing Group L (0.25% levobupivacaine, n = 30) and Group R (0.25% ropivacaine, n = 30). Continuous variables (age, weight, BMI, HR, and MAP) were compared using independent-samples t-tests, and categorical variables (sex and ASA physical status) were compared using chi-square tests. Data are presented as mean ± SD or n (%). Test statistics include t values with degrees of freedom (df = 58) for t-tests and χ² values with df = 1 for 2 × 2 comparisons. Statistical significance was defined as p < 0.05 (two-tailed). No statistically significant differences were observed between the groups (all p ≥ 0.05), confirming baseline comparability. ASA, American Society of Anesthesiologists; HR, heart rate; MAP, mean arterial pressure

Parameter	Group L (n = 30)	Group R (n = 30)	Test statistic	p-value
Age (years), mean ± SD	34.4 ± 9.2	38.9 ± 9.7	t = -1.761	0.09
Weight (kg), mean ± SD	55.0 ± 2.1	53.0 ± 1.1	t = 4.328	0.17
BMI (kg/m²), mean ± SD	22.1 ± 2.3	21.8 ± 2.1	t = 0.506	0.61
Sex (M/F), n (%)	10/20 (33/67)	12/18 (40/60)	χ² = 0.407	0.52
ASA physical status I/II, n (%)	18/12 (60/40)	16/14 (53/47)	χ² = 0.109	0.74
Baseline HR (bpm), mean ± SD	86.7 ± 11.1	84.6 ± 14.8	t = 0.625	0.53
Baseline MAP (mmHg), mean ± SD	87.3 ± 3.5	90.1 ± 6.9	t = -1.984	0.05

Intraoperative hemodynamics

Following induction and intubation, Group R tended to have higher SBP, DBP, MAP, and HR at several intraoperative time points than Group L, with some differences reaching statistical significance, although all values remained within clinically acceptable ranges. No patient in either group required vasopressor support or treatment for hypertension. Immediate postoperative hemodynamic parameters at PACU discharge were comparable between the groups. Table 2 shows the intraoperative hemodynamic parameters.

**Table 2:** Intraoperative hemodynamics HR and MAP at baseline, post-induction, post-intubation, 30 minutes, and PACU discharge in Group L (0.25% levobupivacaine, n = 30) and Group R (0.25% ropivacaine, n = 30). Data are presented as mean ± SD. Comparisons were performed using independent-samples t-tests at each time point, with t values reported for df = 58. Statistical significance was defined as p < 0.05 (two-tailed). An asterisk (*) indicates statistically significant between-group differences at that time point. Significant differences were observed post-induction (HR: t = -1.758, p = 0.04; MAP: t = -4.542, p = 0.04) and post-intubation (HR: t = -1.721, p = 0.03; MAP: t = -2.406, p = 0.02), whereas values were comparable at 30 minutes and PACU discharge (p ≥ 0.06). All hemodynamic values remained within clinically acceptable physiological ranges, and no patient required vasopressor support. HR, heart rate; MAP, mean arterial pressure; PACU, postanesthesia care unit

Time point	Parameter	Group L (mean ± SD)	Group R (mean ± SD)	Test statistic	p-value
Baseline	HR (bpm)	86.7 ± 11.1	84.6 ± 14.8	t = 0.625	0.53
	MAP (mmHg)	87.3 ± 3.5	90.1 ± 6.9	t = -1.984	0.05
Post-induction	HR (bpm)	84.7 ± 14.5	90.6 ± 6.1	t = -1.758*	0.04
	MAP (mmHg)	87.3 ± 3.5	97.1 ± 8.4	t = -4.542*	0.04
Post-intubation	HR (bpm)	89.4 ± 14.3	94.6 ± 4.0	t = -1.721*	0.03
	MAP (mmHg)	82.5 ± 9.9	89.0 ± 9.4	t = -2.406*	0.02
30 minutes	HR (bpm)	83.4 ± 10.8	81.7 ± 9.3	t = 0.661	0.51
	MAP (mmHg)	100.1 ± 11.2	103.5 ± 9.5	t = -1.260	0.21
PACU discharge	HR (bpm)	79.6 ± 4.9	84.4 ± 13.4	t = -1.849	0.06
	MAP (mmHg)	91.1 ± 9.8	94.6 ± 5.2	t = -1.576	0.12

Primary outcome: time from block completion to first VAS ≥4

The duration of analgesia, defined as the time from completion of the BSCPB (performed after induction and before surgical incision, providing a common and consistent starting point independent of individual surgical duration) to the first request for rescue analgesia (VAS ≥4), was significantly longer in Group L than in Group R (549.3 ± 36.3 minutes, approximately nine hours and nine minutes, vs 370.0 ± 59.8 minutes, approximately six hours and 10 minutes; p < 0.001). This corresponds to an absolute prolongation of 179.3 minutes (approximately three hours), representing a 48% increase in the duration of analgesia with levobupivacaine compared with ropivacaine. This magnitude of prolongation is clinically meaningful in the context of thyroid surgery, as it provides more sustained postoperative pain relief during the critical early recovery period and reduces the need for rescue analgesics.

Postoperative pain scores

VAS scores were similar at one hour postoperatively, reflecting good immediate analgesia in both groups. Thereafter, Group L consistently had lower VAS scores than Group R at all assessed time points, with statistically significant differences from four hours onward. Table 3 shows the postoperative VAS scores over 24 hours [15].

**Table 3:** Postoperative VAS scores over the 24-hour period Postoperative pain intensity was assessed using the VAS (0-10, where 0 = no pain and 10 = worst imaginable pain) at one, four, eight, 12, 16, 20, and 24 hours postoperatively in Group L (0.25% levobupivacaine, n = 30) and Group R (0.25% ropivacaine, n = 30). Data are presented as mean ± SD. Between-group comparisons at each time point were performed using independent-samples t-tests, with t values reported for df = 58. A Bonferroni correction for multiple comparisons was applied (p < 0.007 was considered statistically significant for the adjusted α level; 0.05 ÷ 7 = 0.007). Repeated-measures ANOVA demonstrated a significant main effect of group (F = 8.74, p < 0.001, df₁ = 1, df₂ = 58) and a significant time-by-group interaction (F = 4.21, p = 0.03, df₁ = 6, df₂ = 348). Group L showed significantly lower VAS scores than Group R from four hours onward (p ≤ 0.007). An asterisk (*) indicates p < 0.05 (statistically significant). Statistical significance was defined as p < 0.05 (two-tailed). VAS, Visual Analog Scale

Time (hours)	Group L (mean ± SD)	Group R (mean ± SD)	Test statistic	p-value	Interpretation
One	0.00 ± 0.00	0.03 ± 0.18	t = -0.981	0.32	Not significant
Four	0.50 ± 0.50	0.80 ± 0.61	t = -2.074	0.04*	Significant
Eight	1.20 ± 0.48	1.60 ± 0.62	t = -2.833	0.007*	Significant
12	1.30 ± 0.53	1.90 ± 0.88	t = -3.143*	0.002*	Highly significant
16	1.37 ± 1.03	2.50 ± 0.73	t = -4.891*	<0.001*	Highly significant
20	1.80 ± 0.85	2.70 ± 0.45	t = -4.721*	<0.001*	Highly significant
24	2.10 ± 0.95	3.20 ± 1.15	t = -4.021*	<0.001*	Highly significant

Rescue analgesia requirements

The proportion of patients requiring at least one dose of rescue analgesia in the first 24 hours was significantly lower in Group L than in Group R (12/30 (40%) vs 24/30 (80%); relative risk, 0.50; 95% CI, 0.32-0.79; p = 0.002), indicating a substantial reduction in rescue analgesic requirements with levobupivacaine. Among patients who required rescue analgesia, both the mean number of paracetamol doses and the total 24-hour paracetamol consumption were significantly lower in Group L than in Group R (mean number of doses: 1.2 ± 0.6 vs 2.3 ± 1.1, p < 0.001; total 24-hour consumption: 2.0 ± 1.2 g vs 4.6 ± 2.1 g, p < 0.001). Notably, no patient in either group required rescue morphine during the 24-hour observation period, underscoring the adequacy of the analgesic regimen in both groups and the superior opioid-sparing effect associated with levobupivacaine.

Intraoperative opioid consumption

Intraoperative opioid consumption was comparable between the two groups, with no significant difference in total fentanyl-equivalent doses (Group L: 285 ± 42 μg vs Group R: 298 ± 51 μg; p = 0.21). Given that intraoperative fentanyl (a short-acting opioid with a clinical duration of action of approximately 30-60 minutes) was administered only intraoperatively and was comparable between groups, it is pharmacologically implausible that this exposure meaningfully influenced pain scores or analgesic requirements from four hours postoperatively onward. Therefore, the between-group differences in postoperative analgesia are more plausibly attributable to the differing pharmacological properties (duration of sensory block) of levobupivacaine vs ropivacaine rather than to residual intraoperative opioid effects.

Adverse events and safety

No patient in either group experienced clinical evidence of local anesthetic systemic toxicity, hematoma, respiratory compromise, or block-related neurological complications, and the overall safety profile was excellent in both groups. PONV occurred in one of 30 patients (3.3%) in Group L and two of 30 patients (6.7%) in Group R (p = 0.05). Sore throat was reported in three of 30 patients (10%) in Group L and four of 30 patients (13.3%) in Group R (p > 0.05). There were no cases of postoperative hoarseness, recurrent laryngeal nerve injury, hematoma requiring intervention, local anesthetic systemic toxicity, unplanned ICU admission, or extended hospital stay beyond 24 hours in either group. All 60 patients were successfully discharged within the planned 24-hour postoperative period.

## Discussion

Overall interpretation

This prospective observational comparative study indicates that levobupivacaine provides significantly better postoperative analgesia than ropivacaine when used for BSCPB in patients undergoing thyroidectomy under general anesthesia [19]. The primary outcome showed a markedly longer duration of effective analgesia with levobupivacaine (approximately nine hours) compared with ropivacaine (approximately six hours), in line with prior evidence that levobupivacaine produces a longer-lasting peripheral nerve block than ropivacaine [11]. This prolonged effect translated into consistently lower pain scores from four to 24 hours postoperatively and a significant reduction in both the proportion of patients requiring rescue analgesia and total 24-hour paracetamol consumption in the levobupivacaine group, supporting a clinically meaningful analgesic and opioid-sparing advantage for levobupivacaine in thyroid surgery [11,19].

Pharmacological basis for the differences

The superiority of levobupivacaine observed in this study is pharmacologically plausible and consistent with the known physicochemical properties of the two agents. Levobupivacaine, the S(-)-enantiomer of bupivacaine, is slightly more lipophilic and more extensively protein-bound than ropivacaine, which is associated with greater tissue affinity, higher local drug concentrations, and prolonged residence time at the nerve, thereby extending the duration of sensory block [11,12,21]. A PRISMA-compliant meta-analysis by Li et al. demonstrated that levobupivacaine provides a significantly longer duration of block and sensory block and a lower incidence of postoperative rescue analgesia compared with ropivacaine in peripheral nerve blocks at various anatomical sites [11]. These pharmacokinetic and pharmacodynamic differences explain the approximately 48% prolongation of effective analgesia observed with levobupivacaine in this cohort and are consistent with the direction and magnitude of effects reported in previous peripheral nerve block studies [12,21].

Mechanistically, the greater lipophilicity and protein binding of levobupivacaine slow its diffusion away from the nerve and delay systemic vascular uptake, prolonging local anesthetic residence time at the axonal sodium channels and thereby extending the duration of conduction block. In contrast, ropivacaine’s lower lipophilicity favors faster diffusion and vascular clearance, along with preferential blockade of small, poorly myelinated C-fibers (nociceptive) over larger A-beta fibers (motor/proprioceptive), which shortens sensory block duration relative to levobupivacaine while preserving its favorable motor-sparing profile [11,12,21]. Clinically, this mechanistic distinction suggests that levobupivacaine may be prioritized for BSCPB when prolonged sensory analgesia is the primary goal, whereas ropivacaine’s differential block may be preferred when early motor recovery is prioritized.

Relation to previous clinical evidence

Previous comparative trials and meta-analyses of peripheral nerve blocks have consistently reported longer sensory block duration and marginally better analgesia with levobupivacaine than with ropivacaine [11,12]. Li et al. concluded that levobupivacaine is more potent than ropivacaine in peripheral nerve blocks and provides longer-lasting anesthesia, with fewer patients requiring rescue analgesia [11]. Our findings extend and confirm this pattern specifically for BSCPB in thyroidectomy, a high-volume procedure where effective, sustained postoperative analgesia is particularly important [12,19]. At the same time, recent randomized work using 0.25% ropivacaine with adjuvants in BSCPB has shown that ropivacaine-based blocks can provide adequate, safe analgesia with stable hemodynamics, reinforcing that ropivacaine remains an acceptable agent even though levobupivacaine appears superior in terms of duration and rescue requirements [12,19].

Role of BSCPB in thyroidectomy

The present study also reinforces the established role of BSCPB as an integral component of multimodal analgesia for thyroidectomy. A recent systematic review and meta-analysis including 31 studies and 2,273 patients found that BSCPB significantly reduced post-thyroidectomy opioid consumption, prolonged the time from block completion to first VAS ≥4, and lowered 24-hour pain scores and PONV compared with no block or placebo [9]. Another meta-analysis focused on thyroid and parathyroid surgery similarly demonstrated that BSCPB prolongs time to first rescue analgesia and reduces total analgesic requirements in the first 24 hours [21]. Prospective and randomized studies have further shown that BSCPB, whether landmark-guided or ultrasound-guided, improves postoperative pain control and reduces systemic opioid use without increasing complications [5,12,22]. In the present cohort, both levobupivacaine and ropivacaine provided very low VAS scores in the immediate postoperative period, confirming the intrinsic effectiveness of BSCPB as a regional technique; however, levobupivacaine maintained effective analgesia for substantially longer, which is particularly advantageous in thyroidectomy, where pain may impact swallowing and neck mobility well beyond the PACU stay [5,9,19,20,21].

Hemodynamic implications

Hemodynamic observations in this study are consistent with the analgesic differences rather than suggesting intrinsic cardiovascular effects of either drug. Prior work has shown that BSCPB attenuates nociceptive input from the surgical field, thereby improving intraoperative hemodynamic stability and reducing intraoperative opioid requirements during thyroid surgery [5,22]. In our series, both groups exhibited excellent overall hemodynamic stability, and no patient required vasopressor support, indicating that the block technique was safe and effective [19,20]. Slightly higher intraoperative blood pressure and HR values in the ropivacaine group are most plausibly attributable to a relatively weaker or shorter-acting block, leading to greater nociceptive input during surgical stimulation, rather than a direct pharmacological effect of ropivacaine [5,19,20]. This finding is important because it confirms that the analgesic advantage of levobupivacaine was not achieved at the expense of hemodynamic compromise.

Safety and tolerability

The safety profile of both agents in this study was excellent, echoing the broader regional anesthesia literature. Reviews and comparative studies have shown that levobupivacaine and ropivacaine both possess more favorable cardiotoxicity and neurotoxicity profiles than racemic bupivacaine and are associated with very low rates of local anesthetic systemic toxicity when recommended doses are adhered to [22,23]. Clinical studies of BSCPB in thyroid surgery have reported low rates of block-related complications, hematoma, airway compromise, or neurologic deficits when the block is performed by trained clinicians using appropriate techniques and volumes [5,9,21,22]. In the present cohort, no serious adverse events, systemic toxicity, or block-related complications were encountered in either group, and all patients were discharged within the planned 24-hour postoperative period, underscoring that both levobupivacaine and ropivacaine are safe choices for BSCPB in a busy government teaching hospital setting [5,19,22]. This is particularly relevant in high-volume public institutions, where simple, reproducible techniques with good safety margins and minimal adverse effects are essential for sustainable practice.

Clinical and enhanced recovery after surgery (ERAS) implications

The findings of this study have direct implications for perioperative pain management in thyroid surgery within multimodal analgesia and ERAS pathways. Contemporary guidelines from major pain and anesthesia societies emphasize multimodal, opioid-sparing strategies and support procedure-specific regional techniques where evidence demonstrates benefit [9]. Meta-analytic data now clearly support BSCPB as a procedure-specific regional technique for thyroidectomy, given its demonstrated reductions in opioid consumption, pain scores, and PONV [9,21]. Within this framework, a levobupivacaine-based BSCPB appears to be an optimal regional component, providing sustained pain relief over the critical postoperative period with minimal adverse effects and reduced need for rescue analgesia [9,11,19]. For ambulatory thyroid surgery, where patients are frequently discharged four to six hours after surgery, the extended time from block completion to first VAS ≥4 associated with levobupivacaine is particularly advantageous because effective pain control persists well beyond discharge, allowing patients to transition to the home environment with greater comfort, minimal early opioid requirements, and potentially improved satisfaction and functional recovery [9,21].

Collectively, when these data are interpreted alongside existing pharmacologic and clinical evidence, levobupivacaine emerges as a rational preferred long-acting local anesthetic for BSCPB in thyroidectomy under general anesthesia, particularly in high-volume centers implementing multimodal analgesia and ERAS protocols. Future randomized controlled trials directly comparing levobupivacaine and ropivacaine in BSCPB for thyroid surgery, with larger sample sizes and standardized patient-reported outcome measures, will be valuable to further validate these findings and refine dose and technique recommendations [9,11]. In practical ERAS terms, these findings suggest that incorporating levobupivacaine-based BSCPB into standardized thyroidectomy order sets and discharge criteria could allow earlier removal of IV opioid infusions, earlier resumption of oral intake and mobilization, and more predictable same-day or next-morning discharge planning. Future ERAS protocol studies could prospectively track length-of-stay and readiness-for-discharge metrics alongside analgesic outcomes to quantify this benefit.

Study strengths

This observational comparative study has several methodological strengths: (1) prospective design with systematic data collection; (2) blinding of postoperative pain assessors to group allocation; (3) standardized BSCPB technique performed by experienced practitioners; (4) comprehensive outcome assessment, including pain, hemodynamics, and safety; (5) complete follow-up with no patient dropouts; (6) use of validated pain assessment tools (VAS); and (7) detailed documentation of adverse events.

Study limitations

Certain limitations should be acknowledged: (1) This was an observational rather than randomized study, and allocation was sequential rather than random, which could introduce selection bias; a randomized controlled design would enhance the robustness and causal validity of future comparisons and is a priority for subsequent work. (2) The technique used was landmark-guided rather than ultrasound-guided, which may differ from contemporary practice in some centers, although it reflects standard practice in many resource-constrained settings; dedicated phrenic nerve function testing (e.g., diaphragmatic ultrasound) between the first and second side injections was not performed, although continuous pulse oximetry, respiratory rate monitoring, and clinical observation were maintained throughout, and no clinical evidence of hemidiaphragmatic paresis was observed. (3) The study was conducted at a single institution and may not be generalizable to all settings. (4) The study population was limited to ASA I-II patients aged 20-55 years with BMI 18-30 kg/m², so results may not apply to higher-risk or more extreme populations. (5) Blinding of the treating anesthesiologist was not possible due to the nature of the intervention. (6) Follow-up was limited to 24 hours, which captures the critical immediate postoperative period but does not address longer-term outcomes or pain trajectories beyond this period. (7) The sample size, while adequate for the primary VAS-based efficacy outcome, was not powered to detect differences in less common adverse events, and the safety comparisons reported should be interpreted as preliminary and hypothesis-generating. Additionally, plasma concentrations of the local anesthetics were not measured, which would have provided mechanistic insights into the pharmacokinetic differences between the two agents.

## Conclusions

This prospective observational comparative study demonstrates that 0.25% levobupivacaine provides significantly longer and more effective postoperative analgesia than 0.25% ropivacaine when used for BSCPB in patients undergoing thyroidectomy under general anesthesia. Levobupivacaine was associated with a markedly longer time from block completion to first VAS ≥4 (549 minutes vs 370 minutes; p < 0.001), lower postoperative pain scores at all assessed time points from four to 24 hours, a reduced proportion of patients requiring rescue analgesia (40% vs 80%), and a 57% reduction in total 24-hour paracetamol consumption. Both local anesthetics exhibited an excellent and comparable safety profile, with no serious adverse events, block-related complications, or clinical features of local anesthetic systemic toxicity observed in either group. Based on these findings, levobupivacaine can be recommended as the preferred local anesthetic for BSCPB in thyroidectomy, particularly as part of multimodal analgesia and ERAS protocols in similar institutional settings. Its superior analgesic efficacy and prolonged duration of action make levobupivacaine an attractive choice for optimizing postoperative pain control and enhancing recovery in patients undergoing thyroid surgery.
